# Optimizing breast cancer ultrasound diagnosis: a comparative study of AI model performance and image resolution

**DOI:** 10.3389/fonc.2025.1536365

**Published:** 2025-06-06

**Authors:** Yunqing Yin, Junkui Fang, Wei Zhang, Xinying Shen

**Affiliations:** ^1^ The Second Clinical Medical College, Jinan University, Shenzhen, China; ^2^ Department of Interventional Radiology, Shenzhen People’s Hospital, Shenzhen, China

**Keywords:** artificial intelligence, breast cancer, diagnosis, mammography, ultrasound

## Abstract

**Objectives:**

To determine the optimal combination of artificial intelligence (AI) models and ultrasound (US) image resolutions for breast cancer diagnosis and evaluate whether this combination surpasses the diagnostic accuracy of senior radiologists.

**Materials and methods:**

We systematically compared lightweight (MobileNet, Xception) and dense neural networks (ResNet50, DenseNet121) using three image resolutions (224 × 224, 320 × 320, 448 × 448 pixels). A retrospective cohort of 4,998 patients was divided into training/validation (8:2 ratio, *n* = 3,578) and independent testing sets (*n* = 1,410). Diagnostic performance was assessed via AUC, sensitivity, specificity, and analysis speed, with direct comparisons against senior radiologists.

**Results:**

MobileNet with 224 × 224 input achieved the highest AUC (0.924, 95% CI: 0.910–0.938) and accuracy (87.3%) outperforming senior US (AUC: 0.820, accuracy: 79.1%) and mammography doctors (AUC: 0.819, accuracy: 83.6%) (p < 0.05). After excluding BI-RADS 4c and 5 nodules, the diagnostic efficacy of MobileNet_224 is better than that of senior doctors (p < 0.05), can reduce 60.1% false positives of US, and 46.6% of mammography. MobileNet_224 and MobileNet_320 had the fastest analysis speed.

**Conclusion:**

MobileNet_224 represents a novel, efficient AI framework for breast cancer diagnosis demonstrating superior accuracy and speed compared to both complex AI models and experienced clinicians. This work highlights the critical role of optimizing model architecture and resolution to enhance diagnostic workflows and reduce unnecessary biopsies.

## Introduction

Breast cancer (BC) has emerged as the most prevalent malignancy worldwide and is a leading cause of death among women (1, 2). Surpassing lung cancer, it now accounts for over 2.3 million new cases annually representing 30% of all female cancers and 11.7% of all cancers. This malignancy increasingly affects a younger demographic posing a serious threat to women’s health (3).

Currently, clinical diagnosis of BC incorporates various methods, including palpation, digital mammography (DM), magnetic resonance imaging (MRI), and ultrasound (US). Mammography, while widely used, often suffers from high rates of false positives and negatives, particularly in women with dense breast tissue, leading to missed diagnoses (4, 5). MRI is recommended for high-risk BC patients, but its high cost, false-positive rate, and time intensity limit its use to a supplementary role in mammography. US, an important tool for BC screening, is not constrained by mammary gland tissue types and has been shown to increase BC detection rates by 17% while reducing unnecessary biopsies (6, 7). However, US is limited by its reliance on the acoustic impedance difference in tumor tissues making it challenging to differentiate diagnoses, especially in cases of non-mass BC (8). The operator-dependent nature of US also means that diagnostic outcomes can vary significantly based on the experience of the practitioner (9).

The Breast Imaging Reporting and Data System (BI-RADS) has significantly improved the standardization and accuracy of breast tumor diagnosis (10). However, BI-RADS classification relies on visual recognition, which can miss subtle image features. Thus, there is an urgent need for an objective method that minimizes operator dependence and accurately reflects tumor characteristics for BC screening and diagnosis (11).

Advances in AI-driven breast cancer classification have demonstrated significant potential in reducing diagnostic variability and improving clinical workflows. Recent studies, such as those employing convolutional neural network for ultrasound-based classification (12–14), underscore the feasibility of AI in standardizing diagnoses. Furthermore, ensemble machine learning techniques (15, 16) demonstrate improved accuracy through model aggregation. However, these works often lack systematic comparisons across model architectures limiting insights into optimal computational frameworks. While capsule networks (17) show promise in capturing spatial hierarchies within tumor morphology, their computational inefficiency hinders real-time clinical deployment compared to lightweight CNNs. Lightweight architectures, like MobileNet variants (18, 19) have emerged as efficient alternatives in cancer classification, yet prior investigations rarely explore resolution-specific trade-offs or benchmark against both complex models [e.g., ShuffleNet (20), EfficientNet (21)] and human expertise. Concurrently, multi-resolution approaches for medical image segmentation (22, 23) highlight the importance of scale optimization, though their focus remains isolated from end-to-end diagnostic pipelines.

Our study addresses these gaps by systematically comparing lightweight and dense neural networks across resolutions to identify the optimal AI–image combination for breast cancer detection, while directly benchmarking diagnostic efficiency against senior radiologists—thereby advancing clinical standards through technically validated innovation.

## Materials and methods

### Study population

This retrospective study was conducted following approval from the institutional review board of Shenzhen People’s Hospital, with a waiver for informed consent due to its retrospective nature. The Na-exclusion criteria for this study were as follows:

***Inclusion criteria:*** (1) Breast tumors were detected by US, which were classified as 0, 3, 4a, 4b, 4c, or 5 according to BI-RADS. (2) At least 3.0-mm breast tissue can be displayed around the nodule. (3) No intervention or operation was performed on the nodule to be evaluated before ultrasonic examination. (4) Patients underwent surgery or biopsy within 1 week of ultrasonic data collection and obtained pathological results.

***Exclusion criteria:*** (1) BIRADS 1 and 2; (2) Have a history of breast surgery or intervention; (3) Poor image quality; (4) The clinical data of cases are incomplete, and the pathological results are not tracked.

**Figure 1:**
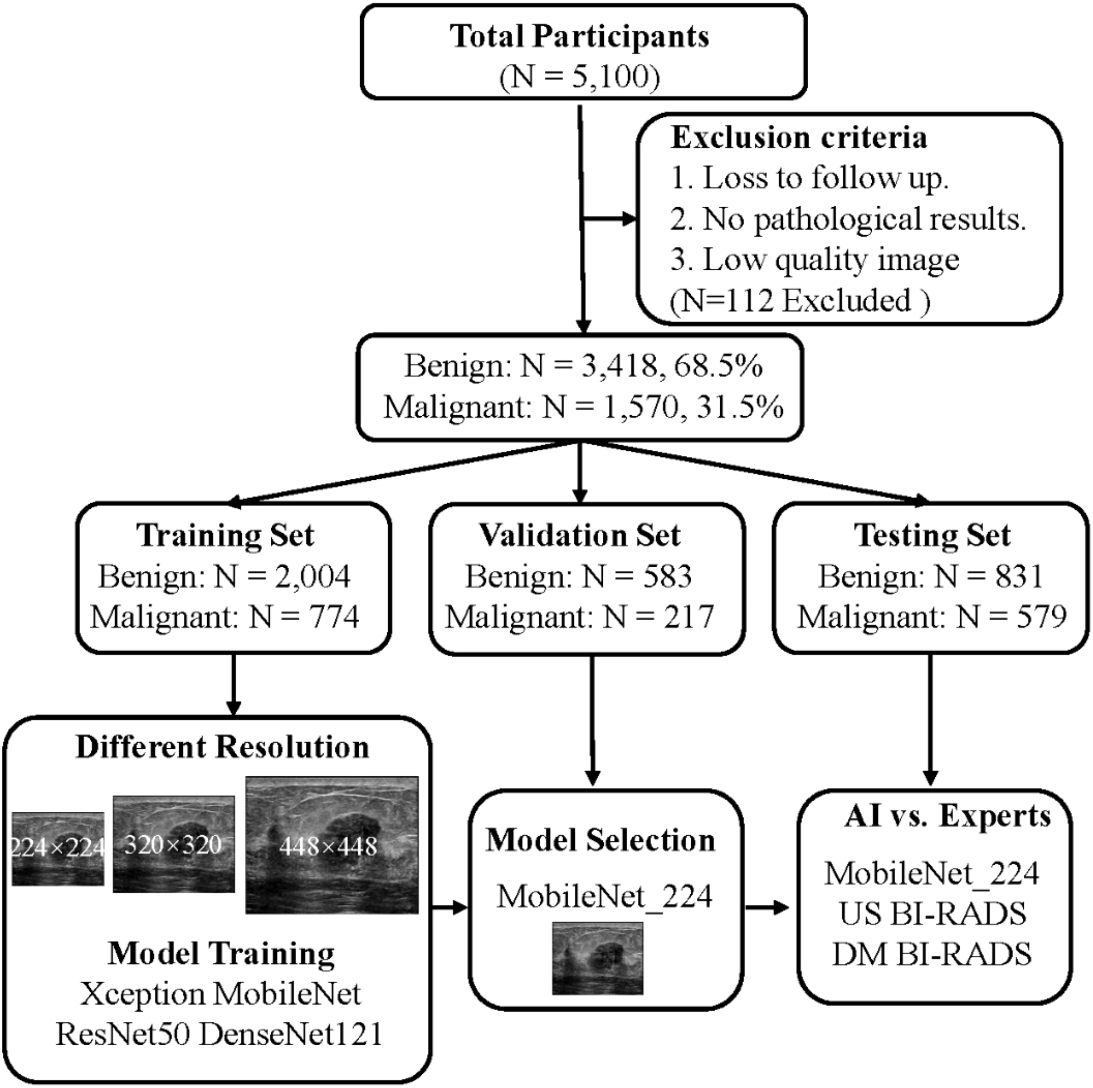
Flow chart and results of this study. The optimal model: MobileNet_224, senior ultrasound doctors, senior mammography doctors. MobileNet_224, MobileNet with 224 × 224-pixel image input; US_BI-RADS, senior ultrasound doctors’ diagnostic results; DM_BI-RADS, senior mammography doctors’ diagnostic results.

In this study, following the inclusion and exclusion criteria, a cohort of 4,998 patients with breast tumors was established. These patients were then randomly divided into the following three groups: a training set, a test set, and an independent validation set. The training and validation sets were allocated in an 8:2 ratio, with the training set comprising 2,778 patients (774 with malignant tumors) and the validation set including 800 patients (217 with malignant tumors). The independent test set consisted of 1,410 patients of whom 579 had malignant tumors (**Table 1**). All patients underwent biopsy or surgical procedures for pathological diagnosis (**Figure 1**).

**Table 1:** Patient information in this study.

Characteristics	Training set		Validation set		Testing set	
	Benign	Malignant	Benign	Malignant	Benign	Malignant
Patients	2,004	774	583	217	831	579
Tumor size (mm)	22.88 ± 9.88		23.17 ± 9.89		22.44 ± 10.07	
Age (year)						
<40	372	106	131	30	154	68
40–49	619	157	203	50	315	135
50–59	602	182	171	52	279	100
60–69	557	150	179	48	338	143
≥70	628	179	116	37	324	133

### Human examination

The US and mammography diagnosis were made by two senior doctors, with more than 10 years of experience in mammography diagnosis, who made the classification diagnosis of benign and malignant tumors under the condition of independent double blind, and gave the BI-RADS classification of tumors. In case of inconsistency, the third chief physician shall be invited for arbitration (**Supplementary Material**). In a comparison of diagnostic performance, BI-RADS classifications 3 and 4A are defined as benign lesions, and 4B, 4C, and 5 are defined as malignant lesions. Diagnostic results from ultrasound doctors and mammography doctors are based on the doctor’s experience.

### AI model construction

Model selection was guided by (1) computational efficiency for clinical deployment, (2) prior evidence in medical imaging, and (3) architectural diversity to benchmark lightweight against dense networks. MobileNet and Xception were prioritized for their parameter efficiency and validated performance in resource-constrained tasks. DenseNet121 and ResNet50 served as benchmarks for hierarchical feature extraction.

These models employ architectural innovations like depthwise separable convolutions to minimize computational burden while retaining diagnostic accuracy. Conversely, dense models, like DenseNet121 and ResNet50—known for their complex hierarchical structures (e.g., residual blocks in ResNet50, dense connectivity in DenseNet121)—were included to evaluate their ability to capture nuanced tumor features in ultrasound images. By comparing these fundamentally distinct architectures, we aimed to identify the optimal balance between computational efficiency and diagnostic precision for breast cancer detection. By comparing these models, we aimed to assess which architecture is more effective for the task of diagnosing BC from US images.

**Figure 2:**
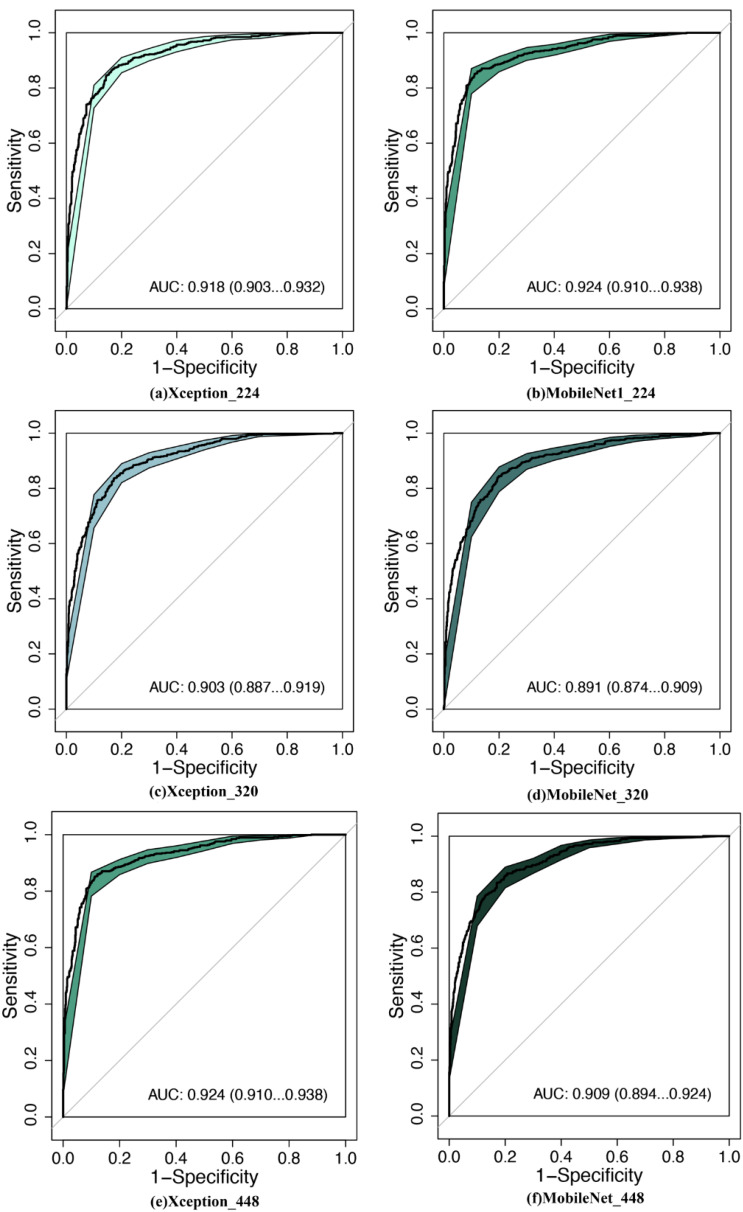
Comparison of diagnostic efficacy between LW-CNNs in the testing set. AUC, area under the curve; 95% CI: 95% confidence interval. **(a)** Xception_224: Xception with 224 × 224-pixel image input, **(b)** MobileNet_224, **(c)** Xception_320, **(d)** MobileNet_320, **(e)** Xception_448, **(f)** MobileNet_448.

We employed the following three different image resolutions: 224 × 224, 320 × 320, and 448 × 448 pixels (illustrated in **Figure 2**). This variation in resolution was intended to examine the impact of image quality on the diagnostic accuracy of the AI models. Higher-resolution images typically provide more detailed information but also require more computational resources to process. Conversely, lower-resolution images are faster to process but may lack some detailed information. Understanding the trade-off between resolution and diagnostic accuracy is crucial for the practical application of AI in medical imaging, particularly in settings where computational resources are limited.

### Training protocol

Models were implemented in TensorFlow 2.5.0, trained on an NVIDIA RTX 3090 GPU, and evaluated on an edge-computing device (Jetson AGX Xavier) to simulate clinical deployment. Images were standardized to 224 × 224, 320 ×3 20, or 448 × 448 pixels. Training employed AdamW optimization (lr = 1e−4) with cosine annealing, and cross-entropy loss weighted for class imbalance (**Supplementary Material**).

To ensure the integrity and non-overlapping nature of our data, we carefully allocated images from the same patient exclusively to one dataset—either the training set or the validation set. This approach was critical to prevent data leakage and ensure that the models were tested on completely unseen data, thereby providing a reliable assessment of their generalizability.

The independent testing set was crucial for evaluating the real-world applicability of the AI models. It consisted of the following three main components: 1) Comparative Evaluation: We assessed the diagnostic effectiveness between different AI models to identify the optimal model and image resolution combination. 2) Comparison with Senior Doctors: The optimal AI model’s diagnostic efficacy was compared with that of experienced senior US and mammography doctors. This comparison aimed to benchmark the AI models against the current gold standard in clinical practice. 3) Exclusion of Certain Tumor Types: We specifically excluded BI-RADS 4c and 5 tumors to focus on challenging cases where AI models could potentially offer the most significant benefit. This step was crucial to understand the potential of AI in improving diagnostic specificity and reducing false positives.

### Statistical analysis

Continuous variable data are expressed as mean ± standard deviation. Categorical variable data are expressed as a percentage. The paired-sample t-test was used to compare the differences within the group. R 3.6.3 was used for the statistical analysis. Diagnostic performance was evaluated using receiver operating characteristic (ROC) curves generated in R 3.6.3 (pROC package). The area under the curve (AUC), reflecting overall discriminative ability, was calculated via the non-parametric DeLong method, with 95% confidence intervals (95% CI) derived from 2,000 stratified bootstrap replicates to account for variability. Sensitivity, specificity, and accuracy were computed from confusion matrices. Statistical significance of AUC differences between models and radiologists was assessed via DeLong’s test (p < 0.05).

## Code availability

The updated code repository and Jupyter notebook was hosted on GitHub—https://github.com/wukaiyeah/ultrasound_breast_malignant_classification.git.

## Results

### Diagnostic performance of AI models vs. radiologists

**Figure 3:**
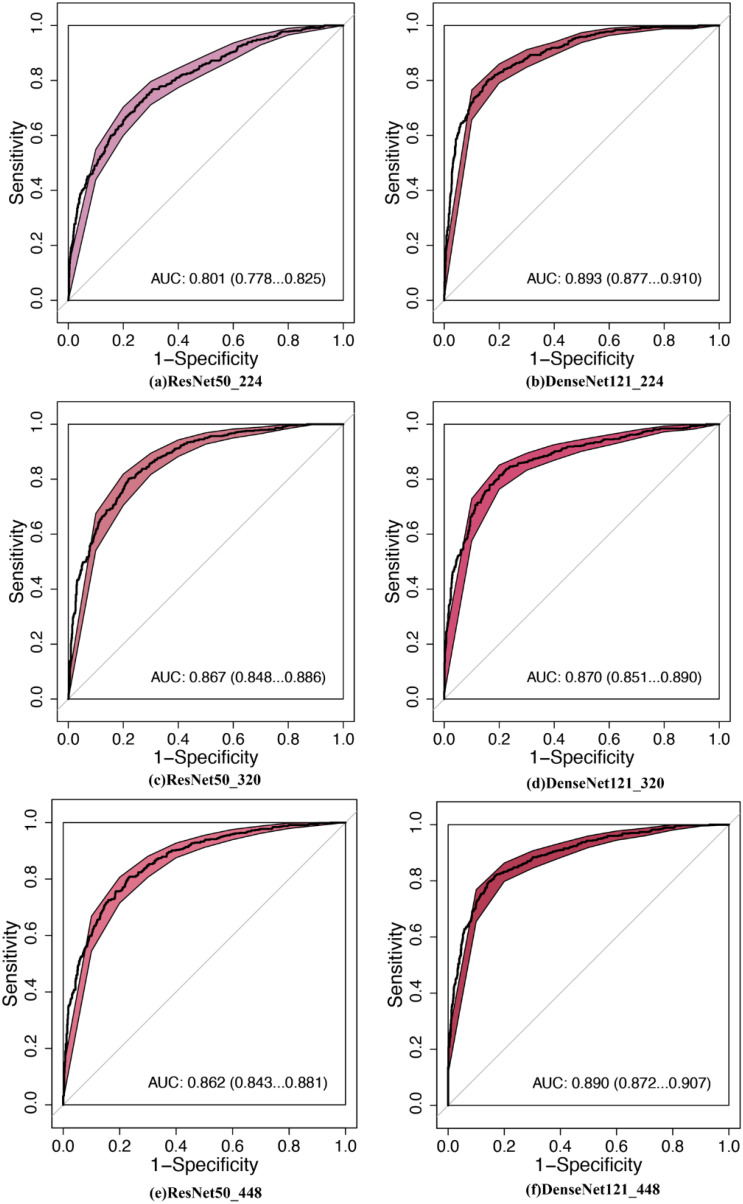
Comparison of diagnostic efficacy between DNNs in the testing set. AUC, area under the curve; 95% CI, 95% confidence interval. **(a)** ResNet50_224: ResNet50 with 224 × 224-pixel image input, **(b)** DenseNet121_224, **(c)** ResNet50_320, **(d)** DenseNet121_320, **(e)** ResNet50_448, **(f)** DenseNet121_448.

MobileNet_224 demonstrated superior diagnostic accuracy compared to both other AI models and senior radiologists. In the independent testing set, MobileNet_224 achieved an AUC of 0.924 (95% CI: 0.910–0.938) significantly outperforming senior ultrasound radiologists (AUC: 0.820, p < 0.001) and mammography specialists (AUC: 0.819, p < 0.001). Its accuracy (87.3%) surpassed radiologists’ performance by 8.2% (ultrasound) and 3.7% (mammography). Dense networks, such as DenseNet121_448, showed lower efficacy (AUC: 0.890; accuracy: 82.8%) highlighting the advantage of lightweight architectures (**Tables 2**, **3**, **Figures 2**, **3**).

**Table 2:** Comparison of the efficacy of AI model in the independent testing set.

Modality	AUC (95% CI)	Cut-off	Sensitivity (%)	Specificity (%)	Accuracy (%)	p-Value
Xception_224	0.918 (0.903–0.932)	0.483	84.6	85.4	85.1	0.230
Xception_320	0.903 (0.887–0.919)	0.290	83.6	82.8	83.1	0.003
Xception_448	0.909 (0.893–0.925)	0.518	82.2	86.5	84.8	0.013
MobileNet_224	0.924 (0.910–0.938)	0.555	85.1	88.8	87.3	NA
MobileNet_320	0.891 (0.874–0.909)	0.209	84.1	80.4	81.9	0.000
MobileNet_448	0.909 (0.894–0.924)	0.670	83.2	83.0	83.1	0.033
ResNet50_224	0.801 (0.778–0.825)	0.214	74.8	71.5	72.8	0.000
ResNet50_320	0.867 (0.848–0.886)	0.466	80.1	78.1	78.9	0.000
ResNet50_448	0.862 (0.843–0.881)	0.319	80.8	76.5	78.3	0.000
DenseNet121_224	0.862 (0.843–0.881)	0.406	80.3	84.0	82.5	0.000
DenseNet121_320	0.870 (0.851–0.890)	0.462	77.9	83.5	81.2	0.000
DenseNet121_448	0.890 (0.872–0.907)	0.460	81.9	83.4	82.8	0.000

AUC, area under the curve, 95% CI, 95% confidence interval; MobileNet_224, MobileNet with 224 × 224-pixel image input, others the same; p, p-value of MobileNet_ 224 compared with other models; NA, not applicable.

**Table 3:** Results of MobileNet_224 and ultrasound/mammography in testing set.

Modality	AUC (95% CI)	Cut-off	Sensitivity (%)	Specificity (%)	Accuracy (%)	p-Value
Model	0.924 (0.910–0.938)	0.555	85.1	88.8	87.3	NA
Ultrasound	0.820 (0.803–0.837)	NA	98.4	65.6	79.1	0.000
Mammography	0.819 (0.799–0.838)	NA	79.7	85.1	83.6	0.000
Model	0.886 (0.854–0.917)	0.467	78.9	86.3	85.2	0.000
US_BI-RADS	0.820 (0.803–0.837)	NA	98.4	65.6	79.1	
Model	0.915 (0.892–0.937)	0.467	84.1	86.7	86.2	0.000
DM_BI-RADS	0.745 (0.714–0.777)	NA	73.9	75.2	74.9	

AUC, area under the curve; AUC, area under the curve; 95% CI, 95% confidence interval; MobileNet_224, MobileNet with 224 × 224-pixel image input; US_BI-RADS, senior ultrasound doctors' diagnostic results; DM_BI-RADS, senior mammography doctors' diagnostic results; p, p-value of MobileNet_ 224 compared with other models; NA, not applicable.

**Figure 4:**
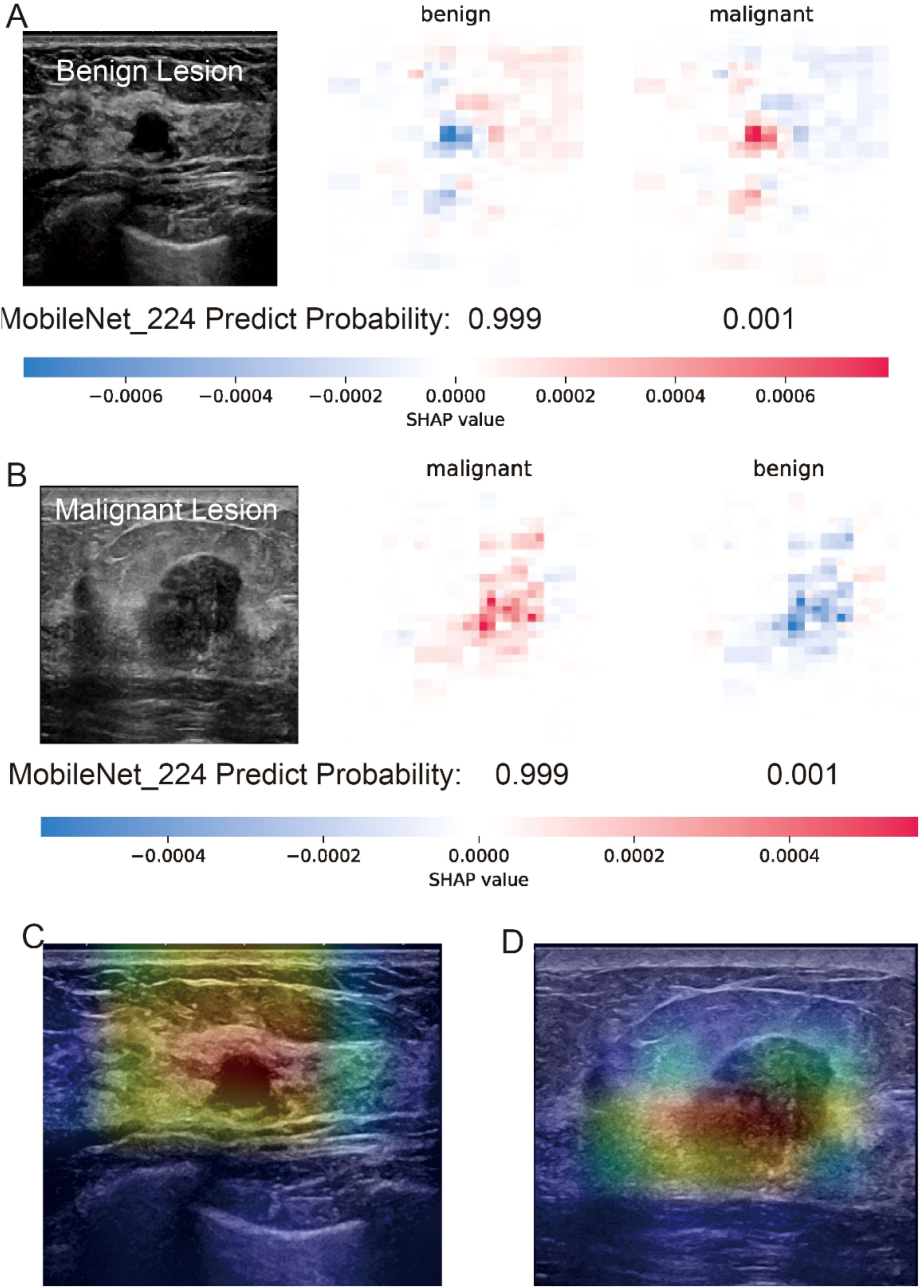
Interpretability analysis of MobileNet_224 predictions for benign and malignant breast lesions. **(A)** Benign lesion: prediction probability (0.999 for benign, 0.001 for malignant) with SHAP values highlighting key image regions contributing to the benign classification. **(B)** Malignant lesion: prediction probability (0.999 for malignant, 0.001 for benign) with SHAP values emphasizing tumor margin irregularity and microcalcifications. **(C, D)** Grad-CAM heatmaps for the benign **(C)** and malignant **(D)** lesions illustrating the model’s focus on clinically relevant anatomical features (e.g., smooth margins in benign vs. spiculated regions in malignant).

The interpretability analysis (**Figure 4**) demonstrates MobileNet_224’s alignment with radiological diagnostic criteria. For benign lesions (A), SHAP values identified smooth margins and homogeneous echotexture as primary contributors to classification, while Grad-CAM heatmaps (C) confirmed focused attention on lesion boundaries. In malignant cases (B), SHAP attributed high malignancy probability to spiculated margins and heterogeneous internal echoes corroborated by Grad-CAM’s emphasis on irregular tumor peripheries (D).

### Impact of image resolution on model performance

Lower-resolution inputs (224 × 224 pixels) consistently outperformed higher resolutions (320 × 320, 448 × 448) across all models. MobileNet_224 achieved the highest AUC (0.924) at 224 × 224, while its performance declined at 448 × 448 (AUC: 0.909). Similarly, Xception_224 (AUC: 0.918) surpassed Xception_448 (AUC: 0.909), despite the latter utilizing more detailed imaging data. This suggests that lower resolutions prioritize clinically decisive features over extraneous textures optimizing both accuracy and computational efficiency (**Table 2**).

### Reduction of false positives and clinical implications

**Figure 5:**
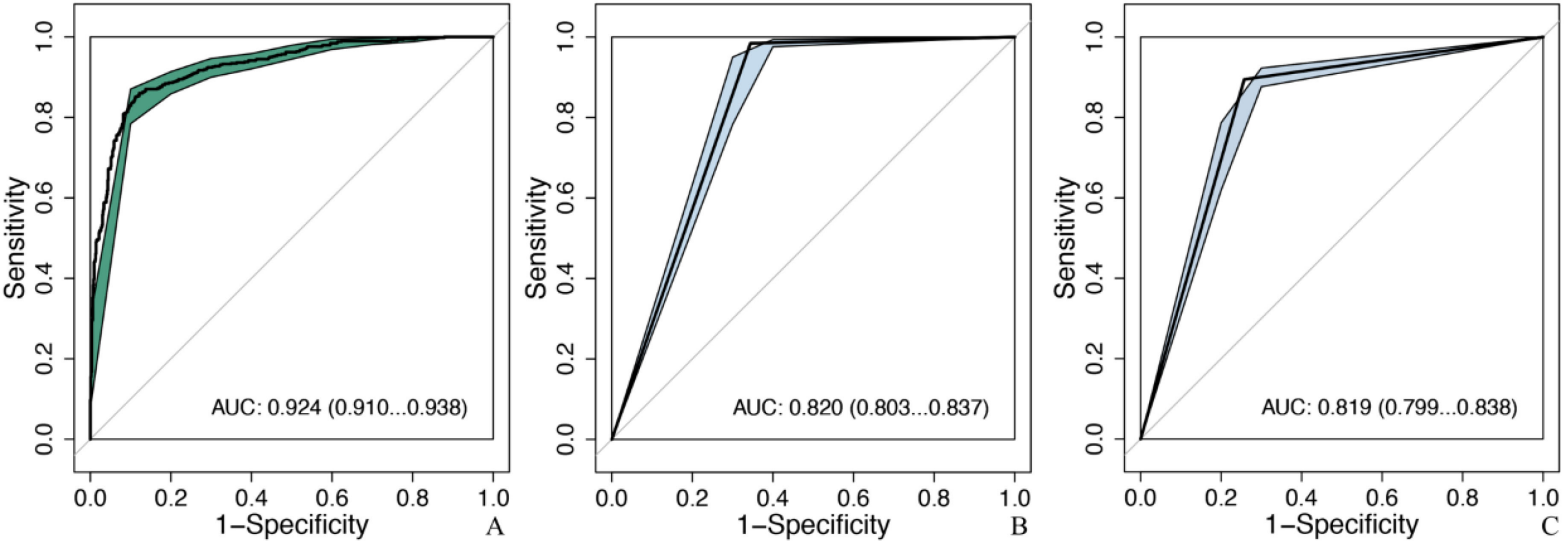
Comparison of diagnostic efficacy between the optimal model and senior doctors in the testing set. MobileNet_224, MobileNet with 224 × 224-pixel image input; AUC, area under the curve; 95% CI, 95% confidence interval. **(A)** The optimal model: MobileNet_224, **(B)** senior ultrasound doctors, **(C)** senior mammography doctors.

**Figure 6:**
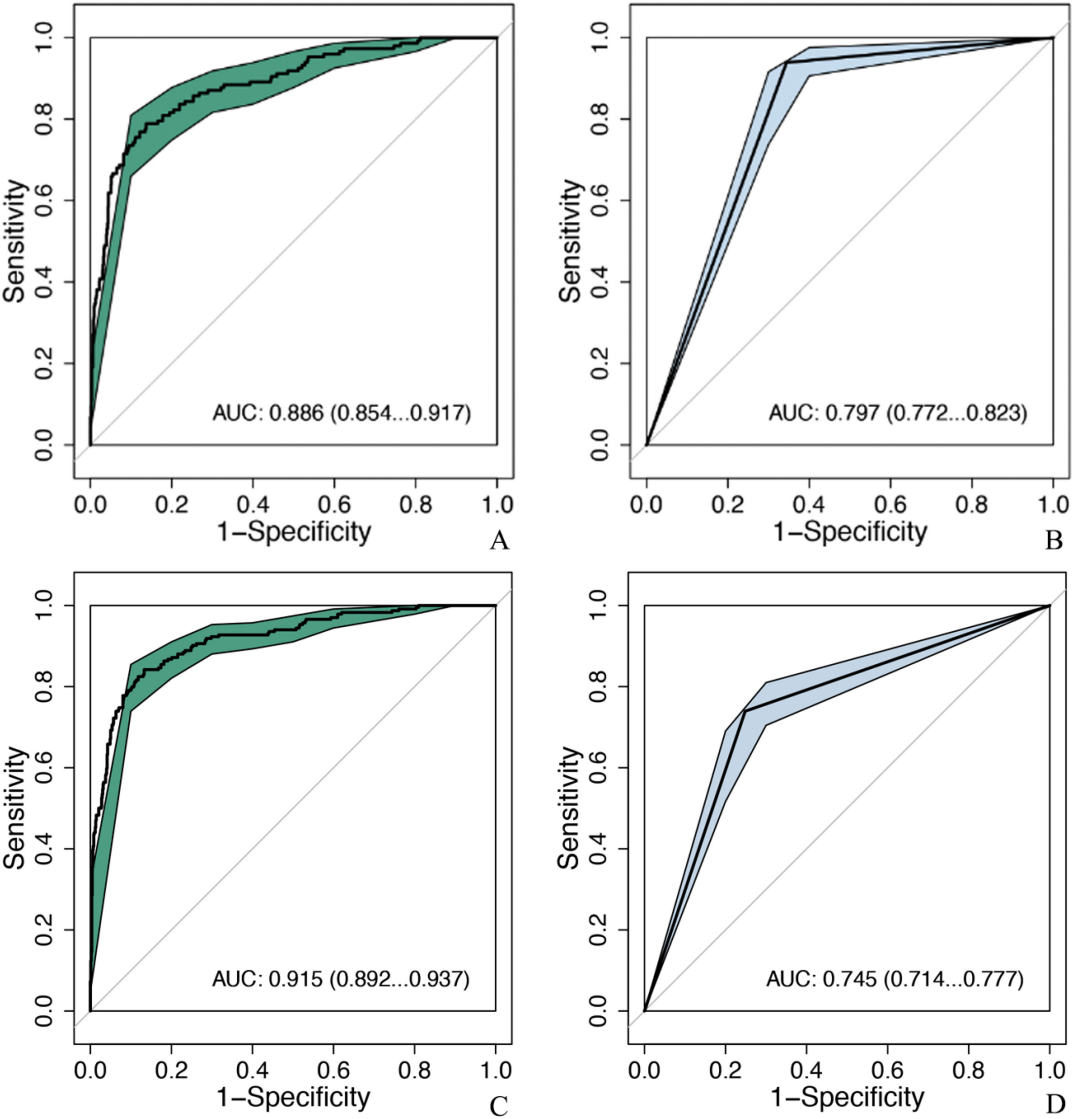
Comparison of diagnostic efficacy between the optimal model and senior doctors after excluding BIRADS 4c and 5 nodules. MobileNet_224, MobileNet with 224 × 224-pixel image input; AUC, area under the curve; 95% CI, 95% confidence interval. **(A)** MobileNet_224, **(B)** senior ultrasound doctors, **(C)** MobileNet_224, **(D)** senior mammography doctors.

MobileNet_224 significantly reduced false-positive diagnoses compared to radiologists: False positives decreased from 286 to 114 cases (60.1% reduction) for ultrasound, and false positives dropped from 204 to 109 cases (46.6% reduction) for mammography. Notably, after excluding BI-RADS 4c/5 cases (high malignancy likelihood), the model maintained superior specificity (88.8% vs. radiologists’ 65.6%, p < 0.001) demonstrating its ability to resolve diagnostically challenging lesions (**Figures 5**, **6**).

### Computational efficiency

MobileNet_224 exhibited the fastest inference speed (0.02 s per image), 3.8× faster than DenseNet121_448 (0.076 s) and 500× faster than manual radiologist review (~10 s per case). This efficiency did not compromise accuracy reinforcing its suitability for real-time clinical workflows (**Table 4**).

**Table 4:** The average time of analyzing a single ultrasound image with different AI models (s).

Modality	224^*^	320^*^	448^*^	Mean ± SD	p-Value
MobileNet	0.019	0.020	0.021	0.020 ± 0.001	0.0004
Xception	0.031	0.034	0.035	0.033 ± 0.002	0.001
DenseNet121	0.075	0.076	0.077	0.076 ± 0.001	0.0001
ResNet50	0.034	0.036	0.037	0.036 ± 0.001	0.0006

Asterisks (*) indicate the image resolution used for model input.

## Discussion

AI has demonstrated remarkable versatility across diverse domains, from anemia detection using palm and conjunctiva images (24–26) to macroeconomic forecasting via time-series models (27). In healthcare, lightweight convolutional neural networks (CNNs) are increasingly applied to resource-constrained tasks, such as MobileNet for diabetic retinopathy screening (28) and Xception for COVID-19 detection (29). Our study extends this paradigm to breast cancer ultrasound diagnosis, where optimizing existing architectures—rather than developing novel models—proves critical for clinical translation.

This study selects four models: Xception, MobileNet, DensNet121, and ResNet50, and 224 × 224-, 320 × 320-, and 448 × 448-pixel image input to explore the accuracy of breast tumors with US images. The results show that MobileNet_224 is superior to the other 11 models and the combination of input images, with an AUC of 0.924 and an accuracy of 87.3%, which are superior to those of senior US and mammography doctors (AUC: 0.820 and 0.819; accuracy: 79.1% and 83.6%).

The application of AI in medical images mainly uses convolutional neural network (CNN) to extract useful information from images. CNN has the following two characteristics: (1) can effectively reduce the dimension of images and (2) can effectively preserve features of images. There are many models derived from this, which are mainly divided into the following two categories: (1) dense neural network (DNN) such as ResNet, DenseNet, and EfficientNet (30, 31); (2) lightweight revolutionary neural networks (LW-CNNs) (32) such as MobileNet, Xception, and ShuffleNet. Large-scale network has a large amount of computation, but the processing speed is slow. LW-CNNs has designed a more efficient network computing method, which not only reduces the number of network layers and parameters but also preserves the performance. It can be used for fast reasoning of embedded and mobile systems. It has a CNN structure with high computational efficiency, adopts point-to-point grouping convolution and channel shuffling, which greatly reduce the amount of computation while maintaining accuracy, and maximize operation speed and accuracy (33, 34). In this study, the diagnostic efficiency of the two LW-CNNs is generally higher than that of the DNNs.

MobileNet (35) was based on a streamlined architecture, and a lightweight deep neural network is constructed using longitudinally separable convolution. Its core idea is that the deep separable convolution replaces the standard convolution and reduces the number of parameters (36, 37). In this study, MobileNet_224 shows the best diagnostic efficiency in different models and images.

Generally, image dimensionality reduction will not affect the final result, such as a picture of 1,000 × 1,000 pixels was reduced to 200 × 200 pixels, which has no obvious impact on the computer recognition results. Among MobileNet with different image resolutions, MobileNet_224 is superior to MobileNet_320 (AUC: 0.891) and MobileNet_448 (AUC: 0.909). The results suggest that MobileNet can still extract the information needed for diagnosis after the image dimension is reduced, which is consistent with the original intention of model design and other studies (38, 39).

A high-resolution image contains more information and larger pixel matrix, but it takes up more memory. In the convolution operation, the large size consumes more computing time than the small size. This study found that the resolution has an impact on the time consumption of the model, and the time consumption of high-resolution model analysis increases, which is consistent with other studies (40). On the contrary, small images consume less computational resources, but may lose some information and may produce misleading results. Therefore, deep learning needs to compromise the contradiction between computational efficiency and recognition accuracy (41). The DNNs, such as DenseNet, ResNet50, and EfficientNetB0, have dense connections between layers and are more memory and time consuming (42). This study shows that DenseNet121_224 takes the longest time in analyzing a single picture, which is 0.07 s, while MobileNet _224 takes less than 0.02 s.

According to BI-RADS classification, Class 0 is a lesion that cannot be determined qualitatively, which has not been diagnosed but has been suspected by doctors, and the possibility of malignancy of BI-RADS 3, 4a, and 4b tumors is less than 2%, 2%–10%, and 10%–50%, respectively. According to BI-RADS 3, follow-up is recommended, and biopsy is recommended for 4a and 4b. If benign tumors can be further screened by AI method, unnecessary puncture and injury can be reduced. A study (43) reported that using the trained AI model to identify benign and malignant breast tumors was higher than the diagnostic level of doctors, the AUC of which were 0.87 (95% CI: 0.79–0.95) and 0.51 (95% CI: 0.50–0.53), respectively. In this study, the AUC of MobileNet_224 [0.886 (95% CI: 0.854–0.917)] is higher than that of senior US doctors [0.820 (95% CI: 0.803–0.837]]. Compared with senior mammography, the AUC of MobileNet_224 [0.915 (95% CI: 0.892–0.937)] is higher than that of senior mammography doctors [0.745 (95% CI: 0.714–0.777)]. To further clarify the diagnostic efficiency of AI technology, this study selected breast tumors that are difficult to diagnose using US and mammography for analysis and found that when the cut-off value of MobileNet_224 is 0.467, the diagnostic accuracy is higher than that of senior doctors in US and mammography. The model significantly reduced false positives in both ultrasound (60.1% reduction) and mammography (46.6% reduction), while improving specificity and overall accuracy (AUC increase: 6.6% for ultrasound; accuracy increase: 6.1%). The application of MobileNet_224 demonstrated significant improvements in diagnostic performance. Specifically, the number of false positives in ultrasound (US) imaging was reduced from 286 to 114 cases representing a 60.1% reduction. For mammography, the model increased the AUC and accuracy by 17% and 11.3%, respectively. Furthermore, the model reduced false positives in mammography from 204 to 109 cases, a decrease of 46.6%. These results highlight MobileNet_224’s capability to diagnose early-stage breast cancer (BC), minimize false positives, and reduce unnecessary biopsies. Contrary to the assumption that higher image resolution universally improves diagnostic accuracy, our findings reveal that MobileNet_224 achieves superior performance at a 224 × 224 resolution. This challenges the prevailing trend in medical AI toward computationally intensive high-resolution frameworks.

The use of AUC, sensitivity, and specificity is widely accepted in oncology AI studies, while analysis speed addresses practical deployment needs. By excluding BI-RADS 4c/5 cases (high malignancy likelihood), we specifically tested the model’s ability to resolve ambiguous diagnoses—a key clinical challenge.

Our study has several limitations. First, this study is a single-center and retrospective study. In the future, a multi-center prospective AI study should be carried out to confirm the reliability of the screening model of this study. Second, this study does not distinguish the types of US instruments and equipment, but only analyzes the static US images. The accuracy and reliability of AI technology for video data analysis need to be studied further. Last, our study focused on evaluating existing lightweight models for clinical deployment capability rather than proposing novel architectures, which limits direct comparisons with cutting-edge frameworks but prioritizes real-world practicality. Future research could expand comparisons to hybrid models, such as CNN-Transformer frameworks, to evaluate their potential for multi-scale feature extraction in breast cancer diagnosis.

## Conclusion

This study systematically evaluates the diagnostic performance of lightweight AI models (MobileNet, Xception) versus dense networks (ResNet50, DenseNet121) across ultrasound image resolutions (224 × 224, 320 × 320, 448 × 448) for breast cancer detection. Using a retrospective cohort of 4,998 patients, we demonstrate that MobileNet_224, despite its computational simplicity, achieves superior clinical utility as follows: 1) Speed–Accuracy Balance: MobileNet_224 processes images in 0.02 s—300× faster than manual review—while maintaining 87.3% accuracy addressing critical workflow bottlenecks. 2) False-Positive Reduction: The model reduces unnecessary biopsies by 60.1% in ultrasound and 46.6% in mammography directly impacting patient outcomes and healthcare costs. 3) Resolution Optimization Framework: Lower resolutions (224 × 224) suffice for accurate diagnosis challenging the need for resource-intensive high-resolution pipelines. These findings advocate for redefining clinical AI benchmarks toward deployment capability rather than theoretical performance offering a pragmatic framework for healthcare translation.

## Data Availability

The raw data supporting the conclusions of this article will be made available by the authors, without undue reservation.
