# Supplementary material for: Optimizing breast cancer ultrasound diagnosis: a comparative study of AI model performance and image resolution
Source: Front Oncol. 2025 Jun 6;15:1536365. doi: 10.3389/fonc.2025.1536365 (PMC12209847; doi:10.3389/fonc.2025.1536365)
Supplement: Supplementary file 1 [file DataSheet1.docx]

**Supplementary Material**

**1 Materials and Methods**

**1.1** **Ultrasound equipment and biopsy needle**

(1) GE LOGIQ E9 color Doppler Ultrasound diagnostic Equipment, GE Company, equipped with ML6-15-D linear array probe.

(2) PHILIPS EPIQ 5 color Doppler Ultrasound diagnostic Equipment, Philips Company, equipped with L12-5 linear array probe.

(3) Mindray Resona 7 color Doppler Ultrasound diagnostic Equipment, Shenzhen Mindray Biomedical Electronics Co., Ltd., China, equipped with L11-3U linear array probe.

(4) Biopsy needle: Japan TSK Company, Acecut 14G.

**1.2 AI related configuration**

**(1) Hardware configuration (**Fig S1**)**

1) Graphics Processing Unit, GPU, NVIDIA SMI 470.57.02.

2) Central Processing Unit, CPU, Intel Core i9-10920X.

3) Memory: DDR4 32G, Kingston Company.

**(2) Software configuration**

1) Anaconda, Individual Edition 64Bit.

2) Python 3.10.3.

3) TensorFlow 2.5.0.

4) Keras deep learning libraries 2.5.0. dev2021032900.

5) Compute Unified Device Architecture, CUDA 11.4.

6) NVIDIA CUDA® Deep Neural Network library, CUDNN 8.2.1.32.

**(3) Model configuration**

1) Xception^1^: https://github.com/kwotsin/TensorFlow-Xception.

2) DensNet^2,3^: CVPR 2017，Best Paper Award,DensNet121，https://github.com/liuzhuang13/DenseNet.

3) MobileNet ^4^: MobileNet v3, https://github.com/Bisonai/mobilenetv3-tensorflow.

4) ResNet^5,6^: ResNet50, https://github.com/sebastianbk/finetuned-resnet50-keras.

**1.****3Ultrasound scanning and diagnosis**

The overall ultrasound examination of breast and surrounding tissues shall be carried out. If the tumor is found, the main evaluation shall be carried out, including the location, size, scope, boundary, edge, shape, internal and posterior echo, calcification and structural changes of surrounding tissues, including skin, pectoral muscle and ligament.

Two chief doctors with more than 10 years of experience in breast ultrasound diagnosis made the classification diagnosis of benign and malignant breast tumors under the condition of independent double-blind, and gave the BI-RADS classification of tumors. In case of inconsistency, the third chief physician shall be invited for arbitration.

**1.4** **Mammography scanning and diagnosis**

Patients were routinely photographed in standing or sitting position. Conventional radiography positions include bilateral mammary glands, internal and external oblique position and cephalopod position. For patients with poor breast display or unable to contain in the conventional posture, the posture can be supplemented according to the location of the focus. If necessary, local pressure photography, magnification photography or local pressure magnification photography can be carried out.

The diagnosis was made by two chief doctors with more than 10 years of experience in mammography diagnosis, who made the classification diagnosis of benign and malignant tumors under the condition of independent double-blind, and gave the BI-RADS classification of tumors. In case of inconsistency, the third chief physician shall be invited for arbitration.

**1.5 Ultrasound image preprocessing**

First, cut the surrounding area of the image in the recorded ultrasound original image, that is, eliminate the equipment related information and patient sensitive information, and only keep the image window.

Next, zoom the image to 224 × 224, 320 × 320 and 448 × 448 pixels. If the part less than the square was filled with black. These scaled images will be randomly divided into input data sets of model training set and testing set. (Fig S2)

**2 Results**

A total of 974 patients with breast tumors were included in this study. They were randomly assigned according to the ratio of training set and testing set: 4:6. The data of training set was 387 cases (174 malignant tumors); The independent testing set data was 587 cases (238 malignant tumors) (Table S1, Fig S3).

**References**

1. Chollet F: Xception: Deep learning with depthwise separable convolutions, Proceedings of the IEEE conference on computer vision and pattern recognition, 2017, pp 1251-1258

2. Huang G, Liu Z, Laurens, et al: Densely Connected Convolutional Networks. arXiv pre-print server, 2018

3. Gao Huang ZL, Laurens van der Maaten, Kilian Q. Weinberger: Densely Connected Convolutional Networks. Proceedings of the IEEE Conference on Computer Vision and Pattern Recognition (CVPR):pp. 4700-4708, 2017

4. Howard AG, Zhu M, Chen B, et al: Mobilenets: Efficient convolutional neural networks for mobile vision applications. arXiv preprint arXiv:1704.04861, 2017

5. He K, Zhang X, Ren S, et al: Deep residual learning for image recognition, Proceedings of the IEEE conference on computer vision and pattern recognition, 2016, pp 770-778

6. He K, Zhang X, Ren S, et al: Deep Residual Learning for Image Recognition. arXiv pre-print server, 2015

**Supplementary Tables and Legends**

**Table S1 Comparison of the efficacy of AI model in the independent validation set**

| **Modality** | **AUC**  **(95%CI)** | **Cut-off** | **Sensitivity (%)** | **Specificity (%)** | **accuracy (%)** | **p**  **value** |
| --- | --- | --- | --- | --- | --- | --- |
| **Xception_224** | 0.914  (0.890-0.937) | 0. 506 | 82.5 | 88.6 | 87.1 | 0.812 |
| **Xception_320** | 0.906  (0.883-0.929) | 0.337 | 82.5 | 87.0 | 85.8 | 0.329 |
| **Xception_448** | 0.901  (0.878-0.924) | 0.500 | 79.7 | 85.1 | 83.6 | 0.106 |
| **MobileNet_224** | 0.916  (0.895-0.937) | 0. 636 | 80.0 | 88.5 | 86.1 | NA |
| **MobileNet_320** | 0.895  (0.871-0.919) | 0.143 | 84.3 | 78.4 | 80.0 | 0.033 |
| **MobileNet_448** | 0.901  (0.877-0.924) | 0.753 | 79.3 | 87.0 | 84.9 | 0.109 |
| **ResNet50_224** | 0.839  (0.808-0.870) | 0.276 | 75.6 | 77.3 | 76.9 | 0.000 |
| **ResNet50_320** | 0.838  (0.808-0.869) | 0.229 | 82.9 | 69.1 | 72.9 | 0.000 |
| **ResNet50_448** | 0.821  (0.788-0.853) | 0.209 | 82.0 | 67.8 | 71.6 | 0.000 |
| **DenseNet121_224** | 0.865  (0.837-0.892) | 0.159 | 82.5 | 73.1 | 75.6 | 0.000 |
| **DenseNet121_320** | 0.889  (0.864-0.914) | 0.244 | 83.9 | 81.0 | 81.8 | 0.008 |
| **DenseNet121_448** | 0.914  (0.893-0.935) | 0.296 | 85.7 | 81.6 | 82.8 | 0.830 |

Note: AUC: Area under the curve, 95%CI: 95%Confidence Interval, MobileNet_224：MobileNet with 224×224 pixels image input, others the same, p: p value of MobileNet_ 224 compared with other models, NA: Not Applicable.

**Supplementary Figures and Legends**


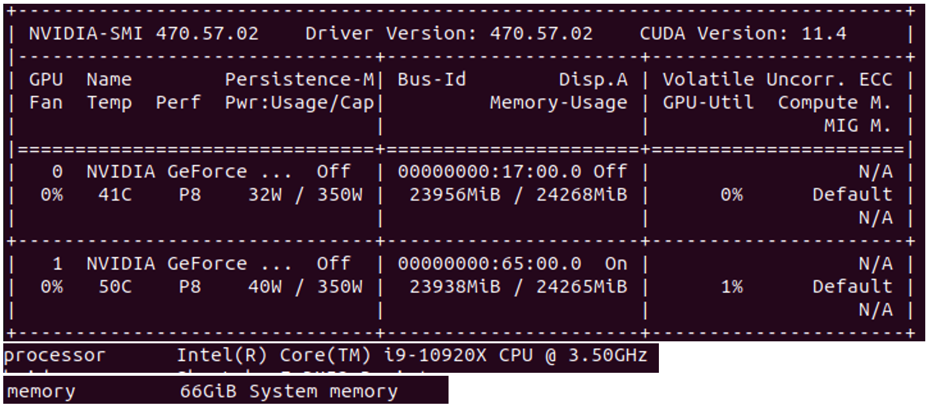

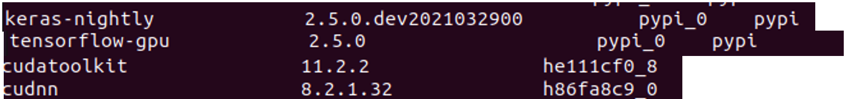


**Figure S1 The main hardware and software configurations.**


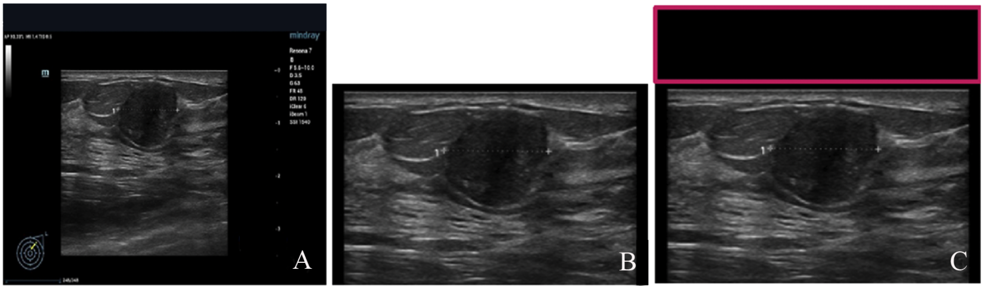


**Figure S2 Ultrasound images preprocessing**

Note: A: original image; B: removed sensitive information; C: the part less than square was filled with black.


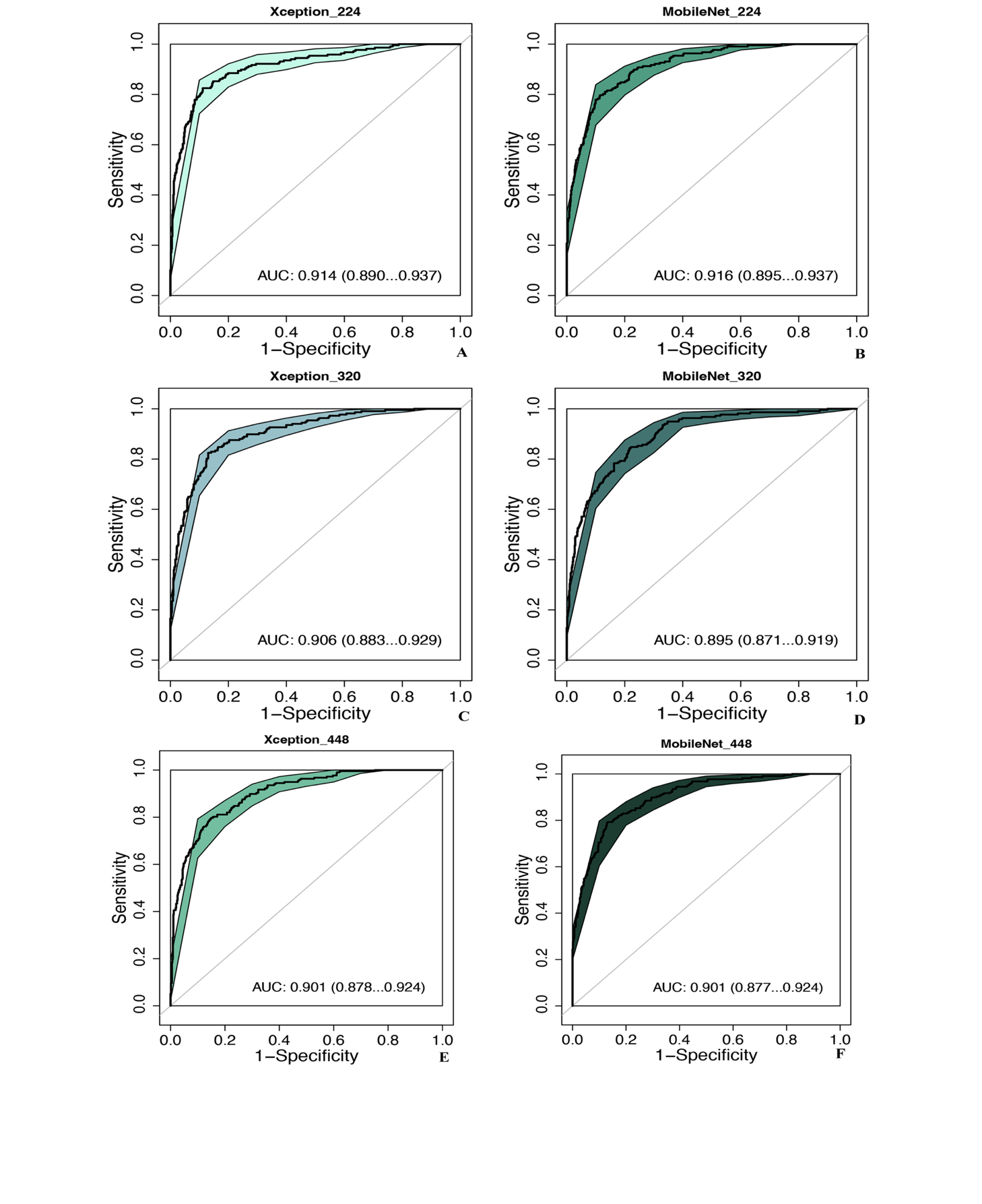
**Figure S3 Comparison of diagnostic efficacy between LW-CNNs in the Validation Set**

**Note:** AUC: Area under the curve, 95%CI: 95%Confidence Interval, A: Xception_224：Xception with 224×224 pixels image input, B: MobileNet_224, C: Xception_320, D: MobileNet_320, E: Xception_448, F: MobileNet_448.


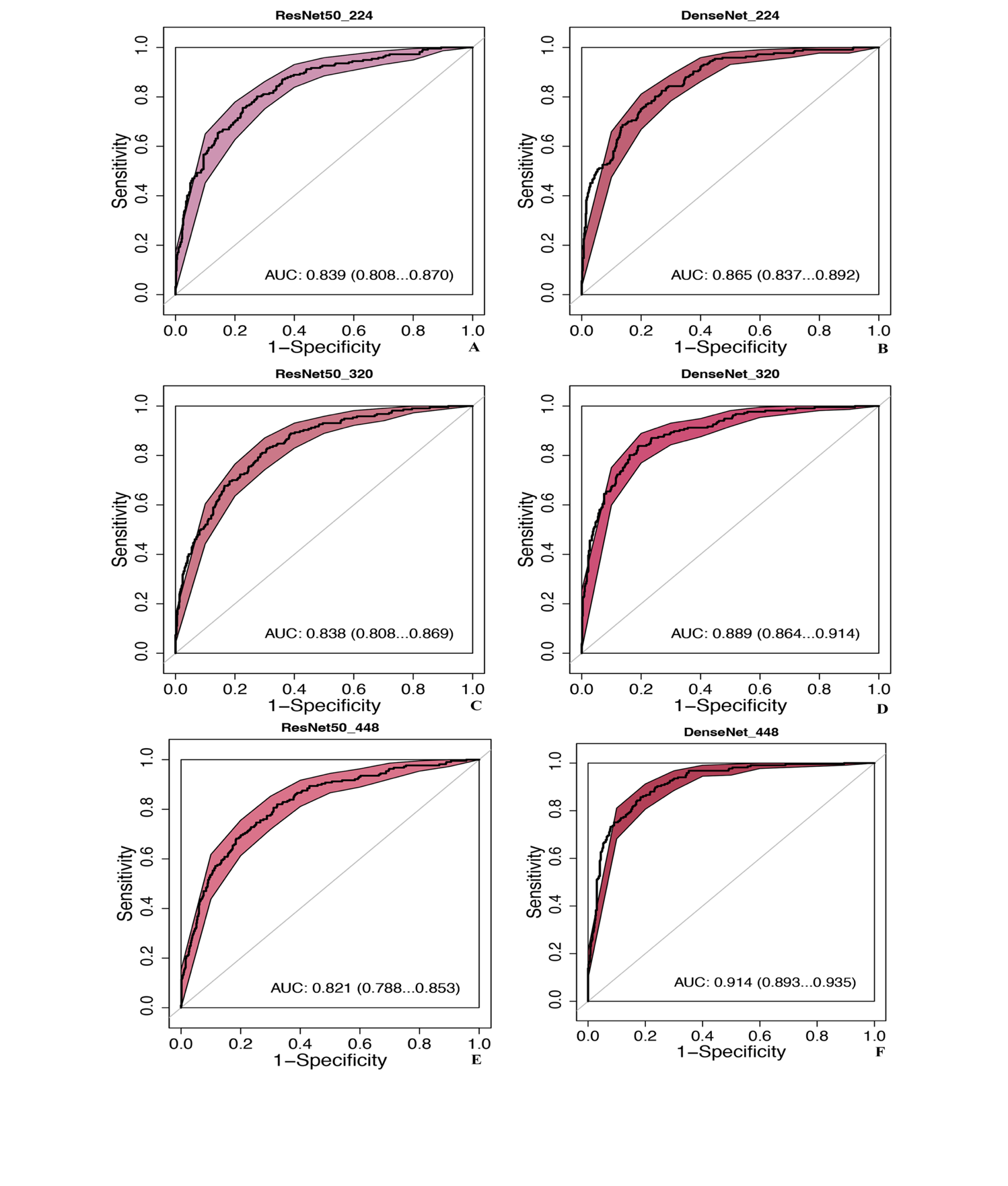


**Figure S4 Comparison of diagnostic efficacy between DNNs in the Validation Set**

**Note:** AUC: Area under the curve, 95%CI: 95%Confidence Interval, A: ResNet50_224：ResNet50 with 224×224 pixels image input, B: DenseNet121_224, C: ResNet50_320, D: DenseNet121_320, E: ResNet50_448, F: DenseNet121_448
